# Dual Upcycling of Olive Leaves for the Biocatalytic Synthesis of Antioxidant Cortisone Derivatives

**DOI:** 10.3390/antiox14070821

**Published:** 2025-07-03

**Authors:** Filippo Marchetti, Irene Gugel, Stefania Costa, Ilenia Gugel, Anna Baldisserotto, Erika Baldini, Stefano Manfredini, Silvia Vertuani

**Affiliations:** Department of Life Sciences and Biotechnology, University of Ferrara, Via Luigi Borsari, 46, 44121 Ferrara, Italy; filippo.marchetti@unife.it (F.M.); irene.gugel@unife.it (I.G.); ilenia.gugel@unife.it (I.G.); anna.baldisserotto@unife.it (A.B.); erika.baldini@unife.it (E.B.); smanfred@unife.it (S.M.); silvia.vertuani@unife.it (S.V.)

**Keywords:** olive leaves, upcycling, circular economy, cortisone, steroids, biocatalysis, bioactive molecules

## Abstract

Bioconversion of cortisone leads to the synthesis of the steroid derivatives 1,9β,17,21-tetrahydroxy-4-methyl-19-nor-9β-pregna-1,3,5(10)-trien-11,20-dione (SCA) and 1,9β,17,20β,21-pentahydroxy-4-methyl-19-nor-9β-pregna-1,3,5(10)-trien-11-one (SCB), which have been identified as biologically active molecules in affections associated with oxidative stress and inflammation, particularly in the skin and eye. To date, the synthesis of SCA and SCB can only be achieved through a biocatalytic approach, following a biotransformation process catalyzed by *Rhodococcus rhodnii* DSM 43960, a synthetic pathway that adheres to the principles of green chemistry. To further enhance the sustainability of this process, this study demonstrated that SCA and SCB can be synthesized by bioconversion in a complex medium derived from a dual upcycling process involving olive leaves (UOLM). By formulating a medium based on olive leaves, a by-product derived from the previously reported biotechnological production of lactic acid, and using a concentration of 10% *v*/*v* UOLM and 1 g/L cortisone at pH 7.5, bioconversion yields of 90 ± 4.5% were achieved, with a predominance of SCB. Investigations into the addition of supplements, such as tryptone, peptone, and corn steep liquor (CSL), to assess potential improvements in yield were conducted, but no significant positive variations were observed. For the first time, bioactive steroids were synthesized from a medium obtained through a dual upcycling process of olive leaves, introducing an innovative method that opens new possibilities for the investigation of a second generation of biosteroids synthesized from lignocellulosic feedstocks.

## 1. Introduction

Olive leaves represent a major by-product of the olive industry, with an estimated annual production of 1 million tons [1], which poses significant challenges regarding their disposal [2]. Given the vast quantities generated and the lack of economically viable disposal routes, olive leaves represent a significantly underutilized resource within the agro-industrial waste stream. Their accumulation can contribute to ecological burdens, such as land overuse and greenhouse gas emissions, if not properly managed [3]. Moreover, olive leaves are known to retain a rich lignocellulosic structure and residual bioactive fractions [2,4] even after initial extraction. This makes them compelling substrates not only for chemical extraction, but also for microbial and enzymatic applications [5,6]. Residual fermentable sugars, such as glucose and cellobiose, further support their value as biotechnological feedstocks [7].

In light of these considerations and building upon our previous study in which olive leaves were utilized for the biotechnological production of second-generation lactic acid [8], the exhausted olive leaf waste resulting from the pretreatment for the lactic acid fermentation process was further valorized. This exemplifies a biorefinery system model where the same biomass is valorized through a multi-stage process. The dual upcycling strategy fosters a circular approach to bioresource utilization, wherein waste material from one fermentation stage serves as the input for the subsequent stage. This not only enhances overall biomass utilization but also diminishes the demand for new raw materials, aligning with the principles of a circular bioeconomy.

**Figure 1:**
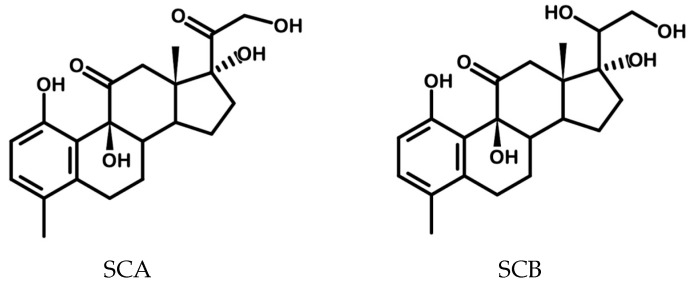
Chemical structures of SCA and SCB.

We recently reported the synthesis of biosteroids from the bioconversion of cortisone using a biocatalytic process driven by *Rhodococcus rhodnii* DSM 43960, this bacterium is able to catabolize steroid molecules by selective uptaking in microbial cells and following the 9,10-*seco* pathway [9], resulting in the following products: 1,9β,17,21-tetrahydroxy-4-methyl-19-nor-9β-pregna-1,3,5(10)-trien-11,20-dione (SCA) and 1,9β,17,20β,21-pentahydroxy-4-methyl-19-nor-9β-pregna-1,3,5(10)-trien-11-one (SCB) recovered in the bioconversion medium (Figure 1).

SCA and SCB are chemically defined by a cyclopentanoperhydrophenanthrene core, with their primary structural modification consisting in the aromatization of ring A into a phenolic moiety, in contrast to the cortisone precursor which retains an aliphatic 3-oxo-4-ene functionality. Beyond the uniqueness of these biomolecules, which, to date, can only be synthesized via biocatalysis, SCA and SCB show promising applications thanks to their potential to exhibit dualistic biological behavior [10]. On one hand, they exhibit anti-inflammatory properties typical of steroidal molecules, while on the other, they display high antioxidant activity, and this identifies them as bioactive molecules with therapeutic potential in a range of diseases that involve both inflammation and oxidative stress such as skin diseases [11] or ophthalmological diseases [12].

Indeed, in a recent study we demonstrated that SCA and SCB can exert protective effect against ARPE-19 and 661W cells from H_2_O_2_-induced oxidative stress, preserving the integrity of tight junction barriers, and maintaining trans-epithelial resistance in ARPE-19 cells after exposure to oxidative stress. These new molecules also improve mitochondrial bioenergetics, identifying these biosteroids as potential candidates for the treatment of eye diseases involving OxInflammation [13], such as age-related macular degeneration, uveitis, retinitis pigmentosa, and diabetic retinopathy [14].

The synthesis of SCA and SCB adheres to a green chemistry methodology, utilizing a bacterial biocatalytic system in an aqueous medium with a near-neutral pH. This process is conducted at low temperatures and avoids the extensive use of chemicals, thereby mitigating high costs and reducing toxicological risks to both the environment and human health. In pursuit of advancing towards a circular economy [15], this study seeks to produce SCA and SCB by substituting the commercially formulated plate count broth (PCB) with a fully upcycled medium derived from olive leaves. Olive leaves underwent a double-upcycling process to yield an upcycled olive leaf medium (UOLM) which was formulated starting from the exhausted solid residue resulting from the pretreatment steps performed on native olive leaves employed for lactic acid production [8]; within UOLM cortisone was bioconverted to SCA and SCB. This represents a significant innovation, as it marks the first instance of olive leaves undergoing a double upcycling process, integrated with the second-generation lactic acid production chain, for the biosynthesis of molecules exhibiting anti-inflammatory and antioxidant properties. These molecules have not previously been synthesized in a medium derived from upcycled feedstock, potentially serving as a foundation for a systematic and exploratory investigation into the synthesis of second-generation biosteroids from lignocellulosic biomass.

## 2. Materials and Methods

### 2.1. Formulation of Upcycled Olive Leaves Medium

**Figure 2:**
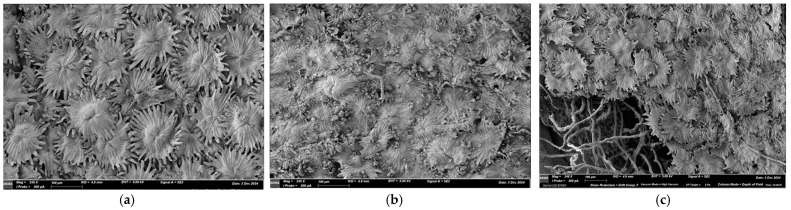
Microscopy of untreated olive leaves (**a**), olive leaves subjected to hydroethanolic extraction and enzymatic treatment (**b**) and to hydroethanolic extraction only (**c**), previously used for lactic acid production.

Native olive leaves underwent a hydroethanolic extraction using 80% ethanol at pH 3, maintained at 60 °C for 4 h. This was followed by an enzymatic saccharification step employing cellulase from *Aspergillus niger* at a concentration of 20 FPU per gram of dried biomass. Liquors deriving from aforementioned pretreatments were used for lactic acid production [3], while solid residue (Figure 2b) was initially dried in a ventilated desiccator at 45 °C for 24 h. Extraction followed the procedure described by Sudjana et al. [16], with minor modifications: 100 g of dried olive leaves was extracted with 1 L of distilled water at 121 °C for 16 min and the solid fraction of the extracted mixture was separated by centrifugation at 5242 RCF for 10 min at room temperature. The supernatant was isolated employing 5000 μL pipette, aliquoted, and frozen at −18 °C until use.

### 2.2. Determination of Olive Leaves Morphology and Physico-Chemical Properties of Olive Leaf Bioconversion Medium

Treated and untreated olive leaves were morphologically analyzed through scanning electron microscopy to identify their morphological characteristics and differences. Microscopic morphological analysis was performed using a Gemini (SEM460) Field Emission SEM (Oberkochen, Germany) under high vacuum (10^−7^ mbar) after coating the sample with a 7 nm layer of gold. The olive leaves-based medium was characterized in terms of the extract color, pH, total suspended solids (TSS), total polyphenols (TPC), ferric reducing antioxidant power (FRAP), and sugar concentration.

Extract color was spectrophotometrically determined by measuring the difference in absorbance at 440 nm and 700 nm [17]; pH was determined using a Basic 20 Crison pH meter (Hach Lange, Barcelona, Spain). TSS was calculated by filtering 5 mL of upcycled olive leaf medium on 0.45 µm cellulose filter and by calculating the difference in weight of the filter before and after filtration, once it has been dried [18]. TPC was measured by referring to the Folin–Ciocalteu test [19] according with method described by Baldisserotto et al. [20]. Briefly, Folin–Ciocalteu reagent was diluted with water in ratio 1:15.8 and 20 μL of sample were allowed to react with the mixture. After 5 min. incubation, 300 μL of a 20% *w*/*v* solution of sodium carbonate were added and samples were incubated in the dark for 90 min. Absorbance was recorded at 765 nm against blank solution. TPC calculated by referring to a calibration curve obtained with gallic acid as reference, evaluated in concentration range of 0–500 ppm. FRAP assay [21] was used to estimate antioxidant power of upcycled olive leaf medium following the method described by Marchetti et al. [22]. In particular, 0.1 M pH 3.6 acetate buffer, 10 mmol/L TPTZ in 40 mmol/HCl, and 20 mmol/L ferric chloride at a 10:1:1 ratio were freshly prepared immediately before analysis and mixed. The execution of the FRAP test was performed by adding 1.9 mL of the FRAP solution and 100 µL of the sample (or solvent for the blank sample). Absorbance at 593 nm was registered after incubation of samples at 37 °C for 10 min. Soluble sugars were determined via HPLC following the method reported by Gugel et al. [8]. Briefly, samples were centrifuged at 6720 RCF for 10 min and filtered with 0.22 μm cellulose acetate filter before injection. Separation was achieved on a Rezex ROA-Organic Acid H^+^ (8%), 300 × 7.8 mm at 60 °C (Phenomenex, Torrance, CA, USA) with 0.6 mL/min isocratic flow where the mobile phase was 0.01 M H_2_SO_4_. The combination of a refractive index detector (Jasco, Oklahoma City, OK, USA) and a UV detector (Jasco, Oklahoma City, OK, USA) at 210 nm was used.

### 2.3. Susceptibility Test to Upcycled Olive Leaf Medium

To determine whether *Rhodococcus rhodnii* was susceptible to toxicity exerted by upcycled olive leaf extract, a disk diffusion test was performed. Briefly, plate count agar (PCA, 1 g/L glucose, 2.5 g/L yeast extract, 5 g/L tryptone, and 15 g/L bacteriological agar) plates were inoculated with a bacterial suspension standardized to a concentration of 1 × 10^5^ CFU/mL, which has been cultured in plate count broth (PCB, 1 g/L glucose, 2.5 g/L yeast extract, and 5 g/L tryptone) for 24 h at 30 °C and 110 rpm. After spreading the bacterial suspension, a sterile cellulose disk with a diameter of 6 mm and 1 mm height was placed at the center, and 50 µL of upcycled olive leaf medium was applied to impregnate the disk at concentrations of 5%, 10%, 20%, 50%, 75%, and 100% *v*/*v*; all dilutions were performed with water. Prior to the deposition of UOLM onto cellulose disks, each extract at every dilution was formulated in triplicate and subjected to a sterilization cycle at 121 °C for 16 min. The plates were incubated at 30 °C for 48 h, inhibition zones were measured, and sterile water was used as a control.

### 2.4. Bioconversion of Cortisone

The bioconversion of cortisone was performed starting with the preparation of a pre-inoculum, where a full loop of *Rhodococcus rhodnii* DSM 43960 (Leibniz Institute DSMZ-German Collection of Microorganism and Cell Cultures GmbH, Braunschweig, Germany), stored as an active culture in PCA, at 4 °C, was transferred into a conical flask (64 × 105 mm diameter × height) containing 20 mL of PCB and capped with cotton plug. The microorganism was allowed to replicate for 24 h at 30 °C in an orbital incubator at 110 rpm (SKI 4 Argolab, Carpi, Italy, square orbiting plane 35 × 35 cm), following the procedures described in our previous works [23,24]. The inoculum was prepared by transferring 50 mL of filtered and sterile upcycled olive leaf medium under the selected conditions into sterile 100 mL nominal volume flasks with a cotton plug. A pre-inoculum volume corresponding to 10% of the working volume (1.5 × 10^6^ CFU/mL) was then added. *Rhodococcus rhodnii* was allowed to grow for 48 h at 30 °C at 110 rpm, after which a cortisone (Merck KGaA, Darmstadt, Germany, ≥95% purity) solution in dimethyl sulfoxide (DMSO) was administered to reach a concentration of 1 g/L in each bioconversion environment. The process was monitored by TLC (silica gel as the stationary phase and ethyl acetate as the mobile phase) and spots were detected phosphomolybdic acid solution. All experiments were performed under sterile conditions, and the results are presented as the mean of at least three experimental replicates.

### 2.5. Effect of Upcycled Olive Leaf Medium and Cortisone Concentration on Biotransformation of Cortisone

The upcycled olive leaf medium was tested at concentrations of 5%, 10%, 20%, 50%, 75%, and 100% *v*/*v* in water, adjusted to pH 7, and sterilized in an autoclave (121 °C, 16 min). Sterilization was performed in 100 mL flasks containing a final working volume of 50 mL. Each culture medium was inoculated with 10% *v*/*v* of *Rhodococcus rhodnii* DSM 43960, previously cultured in PCB under the conditions mentioned above, and allowed to grow for 48 h. To evaluate whether the bioconversion process could be supported by carryover nutrients derived from the preculture medium, a control experiment was performed in which only the bacterial biomass from the preculture was inoculated. The biomass was collected by centrifugation, separated from the residual PCB medium, and washed with sterile saline solution prior to inoculation. A cortisone solution in DMSO was then administrated to each biotransformation medium to reach final concentrations of 1.0 g/L, 1.25 g/L, and 1.5 g/L, and the progress of the bioconversion was monitored qualitatively with TLC and quantitatively with HPLC.

### 2.6. Effect of Initial pH on Biotransformation Performance

At the selected concentration of upcycled olive leaf medium and cortisone, at which the bioconversion reaction showed optimal performance, the effect of the initial pH on the process was studied within the range of 6 to 8 by adjusting the pH of the medium with the addition of 1 M NaOH prior to sterilization. Thus, the biotransformation of cortisone was performed under previously optimized conditions.

### 2.7. Effect of Supplement Addition on Biotransformation Performance

To determine whether the addition of supplements could enhance bioconversion yields, upcycled olive leaf medium was enriched with various supplements. The effects of tryptone and peptone were evaluated at concentrations of 0.7–8 g/L, while corn steep liquor (CSL) was tested at concentrations of 0.07–8 g/L. The pH was adjusted prior to sterilization, and cortisone biotransformation was subsequently performed in enriched media. The bioconversion process was monitored qualitatively by TLC and the products were quantified by HPLC.

### 2.8. Scale-Up, Isolation and Purification of Biosteroids

Under the selected optimal conditions, the cortisone bioconversion was performed by doubling the working volume in sterile 250 mL flasks. The process was carried out using 100 mL of 10% *v*/*v* UOLM at pH 7.5. A pre-inoculum volume corresponding to 10% of the working volume (1.5 × 10^6^ CFU/mL) was added, and *Rhodococcus rhodnii* was allowed to grow for 48 h at 30 °C at 110 rpm; at this time the bacterial concentration was equal to 3.5 × 10^6^ CFU/mL, and 1 g/L of cortisone was administrated, following the procedure outlined in the previous section. Bacterial growth was measured over the biotransformation period and reached values of 7.0 × 10^6^ CFU/mL and 1.1 × 10^7^ CFU/mL after 72 and 96 h, respectively. The bioconversion was monitored via TLC, and upon completion, several aliquots were taken, filtered with 0.22 μm cellulose acetate filters, and subjected to HPLC analysis for quantitative determination of SCA and SCB. After the removal of the bacterial component via centrifugation at 6720 RCF for 10 min (Remi, NEYA 16, Maharashtra, India), each biotransformation was extracted with 3 × 200 mL of ethyl acetate, and the organic extracts were dried over anhydrous Na_2_SO_4_. Dried extracts were concentrated under vacuum to remove the organic solvent, and the crude reaction mixture was purified by preparative column chromatography (20 × 200 mm), using silica as the stationary phase (silica gel 60, 70–230 mesh) and ethyl acetate as the eluent. The eluates were analyzed by TLC, and the fractions containing SCA and SCB were pooled, evaporated, and characterized by ^1^H-NMR (Varian Mercury Plus 400, 400 MHz, Palo Alto, CA, USA) and FTIR spectroscopy (PerkinElmer Spectrum 100, Waltham, MA, USA). FTIR spectra were acquired with ATR method (attenuated total reflectance) scanned from 4000 to 650 cm^−1^, at room temperature, after placing the samples on the crystal, following 4 scans with a resolution of 1 cm^−1^. All the experiments were performed in triplicate.

### 2.9. Analytical Determinations

UV-HPLC analysis was performed following the validated method reported in Marchetti et al. [23]. Briefly, the mobile phase consisted of water (A) acidified with acetic acid and acetonitrile (B), with the following gradient program: 0–4 min 20% B, 4–7 min 30% B, 7–11 min 50% B with isocratic conditions maintained for up to 20 min, 20–20.1 90% B maintained isocratic for up to 25 min, 25–25.1 20% B and isocratic condition to 30 min. Separation was achieved on a stainless-steel C-18 reverse-phase column 250 × 4.6 mm with 5 μm particles at 25 °C (Alltima HP C18 5 μm, Alltech Associates, Inc., Deerfield, IL, USA) and all samples and standards were filtered with a 0.22 µm cellulose acetate filter prior to 20 µL injection. UV detector was set to a wavelength of 289 nm.

## 3. Results and Discussion

In our previous study, olive leaves were used as a substrate for the production of second-generation lactic acid using *Lactobacillus casei*. Prior to fermentation, olive leaves were pretreated through 80% hydroethanolic extraction followed by saccharification with cellulase, allowing the formulation of a liquid medium for fermentation while discarding the solid fraction via filtration [8].

### 3.1. Determination of Olive Leaves Morphology and Physico-Chemical Properties of Olive Leaf Bioconversion Medium

To further valorize the residual cake represented by the solid fraction of olive leaves derived from the lactic acid production bioprocess, the exhausted by-product was dried and initially analyzed by scanning electron microscopy. Micrographs of untreated olive leaves (Figure 2a) and those following extractive and enzymatic treatment (Figure 2b) reveal a substantial morphological difference.

The abaxial regions of the epidermis in treated olive leaves exhibit a significant alteration in the architecture of the trichomes, which appear clearly degraded in several areas when compared to untreated olive leaves and hydroethanolic extracted leaves, which suggests the effectiveness of cellulolytic treatment using cellulase and the potential to recover soluble sugar fractions. These findings are also supported by Fernández et al. [25], where the different morphology of the trichomes in olive leaves before and after treatment with cellulase and pectinase is shown.

To determine whether the olive leaves by-product could be re-extracted for the formulation of novel upcycled media, autoclave aqueous extraction was performed to obtain a bioconversion medium based on the exhausted olive leaves.

Considering the low polyphenol content, as determined by the Folin–Ciocalteu assay (Table 1), and the relatively low antioxidant power of the upcycled olive leaf medium, factors that could inhibit the biocatalytic activity of *Rhodococcus rhodnii*, along with the presence of fermentable sugars such as glucose at a concentration of 6.00 g/L, it was possible to evaluate the potential to extract a residual soluble fraction of saccharides from the already utilized olive leaves to perform the biocatalytic conversion of cortisone.

### 3.2. Disk Diffusion Test

Olive leaf extracts have been extensively studied for their antimicrobial properties against various microorganisms as recently reported by Magyari-Pavel et al. [26,27]; however, there are only a few studies on the antimicrobial activity of plant-based extracts against the *Rhodococcus* genus, and none have focused on olive leaf extracts [28,29].

To the best of our knowledge, there is no scientific evidence in the literature regarding the ability of olive leaf extracts to exert an inhibitory effect on the growth of *Rhodococcus rhodnii*; a bacterial susceptibility test was performed using the disk diffusion method, testing the UOLM at 5%, 10%, 20%, 50%, 75%, and 100% *v*/*v*.

**Figure 3:**
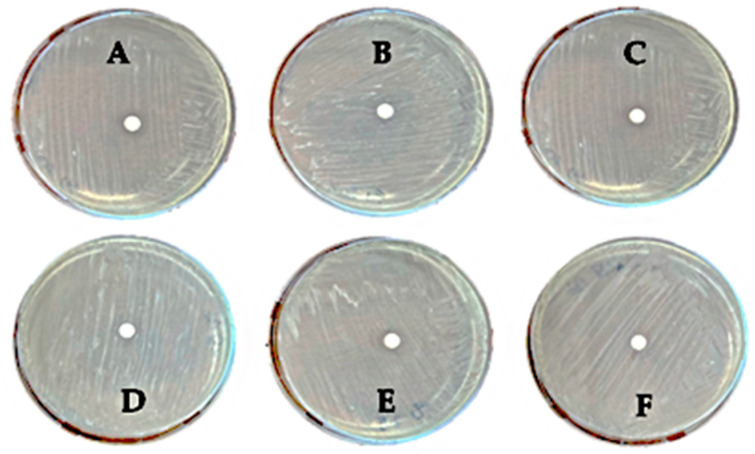
Disk diffusion test for UOLM at concentrations of 5% (**A**), 10% (**B**), 20% (**C**), 50% (**D**), 75% (**E**), and 100% (**F**) *v*/*v* against *Rhodococcus rhodnii* DSM 43960.

As shown in Figure 3A–F, no inhibition zones were observed at any concentration of UOLM, indicating that it did not hinder the growth of *Rhodococcus rhodnii*. This suggests that the upcycled olive leaf extract does not exert toxic activity against this microorganism.

### 3.3. Effect of Upcycled Olive Leaf Medium and Cortisone Concentration on Biotransformation of Cortisone

The bioconversion carried out at UOLM concentrations of 5–100% *v*/*v* in the presence of 1.00, 1.25, and 1.5 g/L cortisone as administered substrate showed the formation of SCA and SCB products, with complete transformation of cortisone into the products only at UOLM concentrations in the 5–20% range, as qualitatively determined by TLC. At higher UOLM concentrations (50–100% *v*/*v*), cortisone was not fully bioconverted into SCA and SCB. No production of SCA or SCB was observed when 10% *v*/*v* of the preculture was inoculated into pure water, as control.

**Figure 4:**
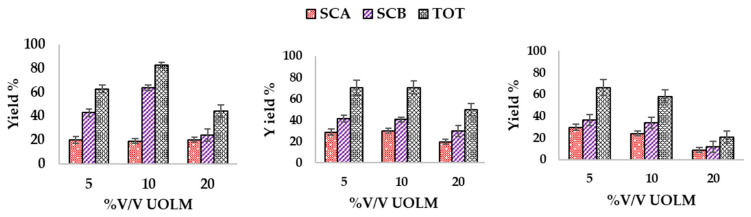
Percentage yields of bioconversions conducted in UOLM at 5%, 10%, and 20% *v*/*v* at 1.00 g/L (**left**), 1.25 g/L (**center**), and 1.50 g/L (**right**) of cortisone administered. Results are expressed as the mean of at least triplicate experiments, with error bars representing the standard deviation.

As demonstrated in Figure 4, increasing cortisone concentration in the administered from 1.00 g/L to 1.50 g/L results in a progressive decrease in the total bioconversion yield. Furthermore, SCA production remains relatively stable without significant variations at a constant cortisone concentration, except when the biocatalytic process is conducted at 1.50 g/L cortisone, where an increase in UOLM concentration from 5% *v*/*v* to 20% *v*/*v* results in a progressive decrease in SCA synthesis.

It can be hypothesized that the complete bioconversion of cortisone may be hindered at higher concentrations of UOLM owing to the release of toxic compounds from lignocellulosic biomass, such as furfural [30] and hydroxymethylfurfural, resulting from high-temperature treatment of olive leaves [31].

In all cases where complete bioconversion of cortisone was achieved, SCB was produced at a higher yield than SCA. This is particularly interesting, as previous studies using the standard plate count broth (PCB) medium—currently considered a reference in our work—have consistently shown significantly higher SCA yields compared to SCB [24]. Under the optimal conditions for UOLM and cortisone concentration, it was observed that at 10% *v*/*v* UOLM with 1 g/L cortisone, the total yield of SCA and SCB reached 83% ± 1.7, with SCA comprising 19% ± 2.1 and SCB 63% ± 2.7.

Based on these results, a UOLM concentration of 10% *v*/*v* and 1 g/L cortisone were selected for further experiments.

### 3.4. Effect of Initial pH of Olive Leaf Upcycled Medium on Biotransformation of Cortisone

**Figure 5:**
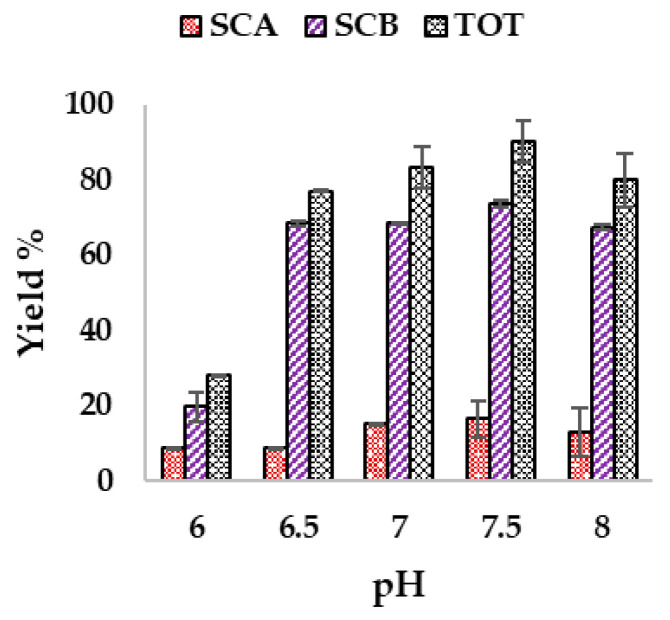
Effect of initial pH of UOLM on the biotransformation of cortisone at 30 °C, 110 rpm, 10% *v*/*v* UOLM, and 1 g/L cortisone.

To determine an effective approach to biotransformation, the initial pH values were analyzed in the range of 6–8, while maintaining a constant temperature of 30 °C and orbital agitation of 110 rpm under conditions of 10% *v*/*v* UOLM and 1 g/L of cortisone. The initial pH at which the biotransformation is carried out can influence the outcome of the process in terms of efficiency [32], particularly because, to the best of our knowledge, no studies have reported the effect of pH on *Rhodococcus rhodnii* DSM 43960. As shown in Figure 5, increasing the initial pH of the medium resulted in a progressive increase in the total yield of the two steroids, primarily driven by SCB synthesis. SCB reached its maximum production at an initial pH of 7.5, where it was produced with a yield of 73.72% ± 0.85. SCA also showed an increase in yield as the pH increased from 6 to 7.5, although the increase was significantly smaller than that of SCB. At pH 7.5, SCA achieved a production yield of 16.4% ± 4.78, which was not substantially different from the yield obtained at pH 7. The pH of 7.5 was therefore selected as the optimum pH for the synthesis of SCA and SCB from cortisone bioconversion, demonstrating the highest total yield (90.13 ± 4.47). The drastic decrease in yield of both biosteroids at pH 6 and the slight decrease at pH 8 could be attributed to the fact that, under non-optimal pH conditions, membrane proteins, multienzyme complexes, and transmembrane transport systems may be less stable, leading to metabolic alterations in the microorganism [32]. It is known that bacteria belonging to the genus *Rhodococcus* are able to grow at an optimal pH that ranges between neutral and slightly alkaline [33] and can express various mechanisms that allow to overcome stress induced by excessively acidic or alkaline cultivation conditions [34]; however, to date, none of these mechanisms have been investigated for the biotechnological applications of *Rhodococcus rhodnii*.

### 3.5. Effect of Supplementation of Olive Leaves Upcycled Medium on Biotransfomation Performances

The roles of tryptone and peptone in biotechnological processes have been well established. These supplements primarily provide nitrogen, amino acids, and growth factors, which can positively influence metabolite production in bioconversion processes; however, they generally incur high costs [35].

With the aim of formulating a fully upcycled medium, corn-steep liquor, whose physicochemical specifications are reported in Table S1, was also tested as a supplement and added to the UOLM to verify its influence on biotransformation performance. CSL is a by-product from corn refining processes and can be utilized in various sectors as an additive in the sustainable biotechnological production of fine chemicals [36].

**Figure 6:**
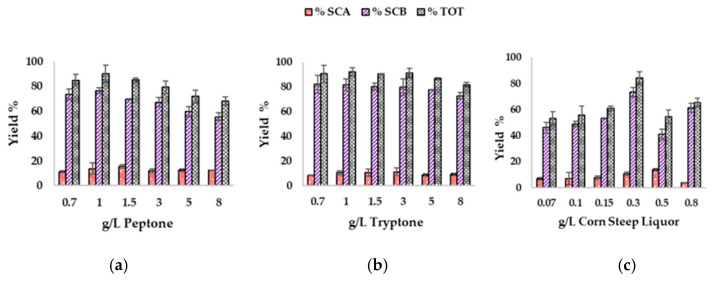
Effects of supplementation with peptone (**a**), tryptone (**b**), and corn-steep liquor (**c**) on biotransformation of 1 g/L cortisone in 10% UOLM at pH 7.5, 30 °C, and 110 rpm.

In the bioconversion of cortisone (Figure 6), where peptone was used as a supplement, the percentage of SCA synthesized increased from 11% ± 0.9 at 0.07 g/L to 16% ± 1.3 at 1.5 g/L of peptone. However, this percentage decreased when higher peptone concentrations were added to the UOLM at 10% *v*/*v*. The concentration of SCB did not show significant variation between 0.07 g/L (74% ± 4.3) and 1.0 g/L (77% ± 2.2) of peptone but declined at concentrations above 1.0 g/L. Under optimal conditions, with 1.0 g/L of peptone added, the total bioconversion yield reached 90% ± 7.0, which does not justify the need for peptone supplementation in the bioconversion medium. This is because UOLM alone, at 10% *v*/*v* and pH 7.5, achieved a comparable total bioconversion yield of 90% ± 4.5.

When tryptone was used as a supplement, the SCA production trend was like the one observed with peptone. In this case, increasing the concentration of tryptone from 0.7 g/L to 3.0 g/L led to an increase in the SCA yield, reaching a maximum of 11 ± 3.0%. However, further increases in the tryptone concentration beyond 3.0 g/L caused the yield to decrease. The synthesis of SCB peaked at a tryptone concentration of 1.0 g/L, with a yield% of 82% ± 4.8. Based on these observations, the total process yield reached its highest value at a tryptone concentration of 1.0 g/L, corresponding to a yield% of 92% ± 3.1. Again, the addition of a supplement such as tryptone does not seem justified when compared to the yields obtained using UOLM alone at 10% *v*/*v* and pH 7.5. Both tryptone and peptone are supplements that introduce additional costs to the process [37,38], which, in this case, do not justify the production of SCA and SCB in relevant quantities compared to using UOLM at 10% *v*/*v* and pH 7.5 alone. This reinforces the notion that UOLM, derived entirely from agricultural waste, serves not only as a cost-effective alternative to synthetic media, but also minimizes the use of chemically defined nutrients. Its successful application validates olive leaf waste as a practical medium component with competitive performance, contributing to more economical and environmentally sustainable bioprocesses.

The addition of CSL as a supplement for cortisone bioconversion in UOLM 10% *v*/*v* at the same concentrations tested for tryptone and peptone completely suppressed or led to an incomplete biocatalytic process. However, subsequent tests showed that a dilution effect on CSL allowed the detection of SCA and SCB via TLC, suggesting that CSL concentrations in the range of 0.7–8.0 g/L were excessive. Therefore, a 1:10 dilution of the supplement was applied, and it was subsequently added to 10% *v*/*v* UOLM at a final concentration tested within the range of 0.07–0.8 g/L. The quantification of SCA and SCB revealed that at every tested concentration of CSL, there was a decrease in bioconversion yield compared to the results obtained with UOLM 10% *v*/*v* at pH 7.5. These findings indicate that CSL cannot be used as a supplement for cortisone bioconversion under selected conditions.

Although CSL is widely used as a supplement, particularly as a nitrogen source, there are reported cases where CSL can negatively affect the performance of biotechnological processes, especially when added at non-optimal concentrations that can alter the carbon-to-nitrogen ratio [39], leading to secondary substrate limitations [40].

### 3.6. Scale-Up of Cortisone Bioconversion, Isolation, Purification and Characterization of the Products

After identifying that the use of peptone, tryptone, and CSL did not result in significantly positive variations in the bioconversion process, it was demonstrated that doubling the operational volume of the process maintained SCA and SCB titers consistent with the trials performed in small batches. The bioconversion yield of SCA in 100 mL of 10% *v*/*v* UOLM at pH 7.5 with 1 g/L cortisone was 17% ± 1.3, while SCB yield was 74% ± 2.4, resulting in a total process yield of 91% ± 2.3, which are in line with the data obtained in previous determination. The specific productivity (Q) in UOLM was 0.0074 and 0.0322 g/L·h for SCA and SCB, respectively; these values are quite different compared to those obtained with the standard medium PCB—in this case the specific productivity was 0.0235 (Q_SCA_) and 0.0096 (Q_SCB_) g/L·h, showing a different behavior. Each batch was extracted three times with 200 mL of ethyl acetate after the bacterial biomass had been removed. The extracts, dried over anhydrous Na_2_SO_4_, were evaporated under vacuum and subjected to double elution on a chromatographic column (silica as stationary phase, ethylacetate as mobile phase). The isolated dried fractions showed two dark white/yellowish solids readily reducible to a fine powder.

Proton NMR spectrum of SCA typically displays diagnostic signals (Figure S1A) which are identified as a doublet at 6.90 ppm (J = 8 Hz) and another doublet at 6.40 ppm (J = 8 Hz), corresponding to the H-C protons in positions 2 and 3 of the phenolic residue, which are indicative of the aromatization of ring A in the cyclopentanoperhydrophenanthrene nucleus of cortisone; both of this signals found correspondence in [24]. Additionally, a singlet signal appears at 9.24 ppm, corresponding to the hydroxyl proton of the phenolic residue, which was reported at 9.22 ppm in the work published by Zappaterra et al. [24]. A singlet at 4.73 ppm is attributed to the hydroxyl proton in position 9, while the hydroxyl proton at position 17 is represented by a signal at 5.41 ppm. Finally, a double doublet at 4.66 ppm (J = 6.6, 5.4 Hz) is associated with the hydroxyl proton on the side chain in position 21 of the steroid nucleus. All aforementioned signals were found in Zappaterra et al. [24], with difference in chemical shift of at least ± 0.02 ppm.

SCB (Figure S1B), which structurally differs from SCA by the presence of a hydroxyl group at position 20 of the side chain, replacing a ketone residue, also shows typical diagnostic signals. These include doublets at 6.86 ppm (J = 8 Hz) and 6.46 ppm (J = 8 Hz) and a singlet at 9.14 ppm, which again are indicative of the formation of the phenolic residue on ring A of the precursor. In comparison to signals reported in [24], doublets were reported at 6.90 and 6.45 ppm, respectively, while the singlet was at 9.15 ppm. Singlets at 4.69 ppm and 3.84 ppm are assigned to the hydroxyl protons at C-9 and C-17 positions (4.68 and 3.85 ppm in Zappaterra et al.), respectively, while the singlet at 4.36 ppm corresponds to the hydroxyl proton at position C-21, reported at 4.38 ppm in Zappaterra et al. [24]. In comparison to SCA, SCB exhibits a diagnostic signal for the hydroxyl group at position C-20 at 4.16 ppm (J = 6.4 Hz), which represents the only structural difference between the two steroids, and which was found at 4.68 ppm in Zappaterra et al. All signals of the biosteroids obtained through bioconversion in UOLM, if compared with the reference data reported, show correspondence [24].

FTIR spectra, performed in normal mode analysis and presented in Figure S2A,B, of SCA and SCB displayed broad bands above 3200 cm^−1^, indicative of O-H group stretching, while sharp peaks at 2931 cm^−1^ were identified as characteristic of C-H stretching. Sharp peaks associated with carbonyl stretching appeared at 1698 cm^−1^ in SCB and at 1709 cm^−1^ in SCA. Both compounds exhibited a sharp medium-intensity peak at 1591 cm^−1^, corresponding to aromatic C-C stretching. Additionally, both biosteroids presented signals in the 1375–1385 cm^−1^ range, attributed to methyl bending vibrations. A sharp peak between 1256 and 1259 cm^−1^ was linked to C-O stretching of the phenolic hydroxyl group, while the C-O stretching of non-phenolic hydroxyl groups appeared between 1046 and 1048 cm^−1^. Lastly, strong, sharp peaks between 750 and 850 cm^−1^ corresponded to out-of-plane C-H bending, confirming the typical molecular structure.

## 4. Conclusions

In this study we successfully synthesized SCA and SCB, biosteroids with dual anti-inflammatory and antioxidant activities, using a medium derived from olive leaves, a lignocellulosic feedstock subjected to a double upcycling process. In formulated UOLM at 10% *v*/*v* and pH 7.5, the biosynthetic process yielded 91% ± 2.3, with SCA at 17% ± 1.3 and SCB at 74% ± 2.4, an outcome that presents a different trend compared to previous findings, where SCA was the predominant biosteroid. In this case, SCB was produced in major quantities, whereas in earlier studies cortisone bioconversion in PCB mainly yielded SCA. The scientific significance of this study lies in demonstrating, for the first time, that SCA and SCB can be produced from lignocellulosic feedstock in high total yields, that the use of an upcycled medium favors SCB synthesis, and that a double upcycling process for olive leaves can effectively build a medium for cortisone bioconversion into SCA and SCB. Importantly, this work marks the first demonstration of a fully upcycled bioconversion medium yielding novel bioactive steroid derivatives thus extending the range of appliable substrate. The implications are both scientific and industrial, suggesting a paradigm shift in how we approach pharmaceutical precursor production—leveraging agro-industrial residues to obtain therapeutically relevant compounds through green biotechnological routes. This study opens the horizon to exploring lignocellulosic biomass for the production of a second generation of biosteroids adopting a sustainable approach, further advancing the circular bioeconomy model.

## Figures and Tables

**Table 1:** Key physical-chemical properties of the upcycled olive leaf bioconversion medium obtained by autoclave extraction at 121 °C for 16 min, using a plant material-to-water ratio of 1:10.

Parameter	Content	SD	Units
pH	4.02	0.01	-
Dry weight	1.27	0.03	mg/mL
Total suspended solids (TSS)	6.79	0.21	mg/mL
Color_(A440-A700)_	1.21	0.00	-
Ferric reducing antioxidant power (FRAP)	3.53	0.04	μmol T/g
Total polyphenols (TPC)	2305.96	108.46	μg GAE/mL
Sugars:			
Cellobiose	0.68	0.00	mg/mL
Glucose	6.00	0.00	mg/mL
Arabinose	0.65	0.00	mg/mL

## Data Availability

The original contributions presented in the study are included in the article/Supplementary Material, further inquiries can be directed to the corresponding author.

## Supplementary Materials

The following supporting information can be downloaded at https://www.mdpi.com/article/10.3390/antiox14070821/s1, Figure S1: ^1^H-NMR spectra of SCA (A) and SCB (B), Figure S2: FTIR spectra of SCA (A) and SCB (B) derived from bioconversion of cortisone in UOLM; Table S1: Chemico-physical specifications of corn-steep liquor.
