# Supplementary material for: Dual Upcycling of Olive Leaves for the Biocatalytic Synthesis of Antioxidant Cortisone Derivatives
Source: Antioxidants (Basel). 2025 Jul 3;14(7):821. doi: 10.3390/antiox14070821 (PMC12291689; doi:10.3390/antiox14070821)
Supplement: Supplementary file 1 [file antioxidants-14-00821-s001.zip › antioxidants-3709291-supplementary.pdf]

Dual Upcycling of Olive Leaves for the Biocatalytic Synthesis of Antioxidant Cortisone Derivatives

Irene Gugel [1†], Filippo Marchetti [1*], Stefania Costa [1*], Ilenia Gugel [1], Anna Baldisserotto [1], Erika Baldini [1†], Stefano Manfredini [1] and Silvia Vertuani [1]

[1] Department of Life Sciences and Biotechnology, University of Ferrara, Via Luigi Borsari, 46 44121 Ferrara, Italy; filippo.marchetti@unife.it (F.M.), irene.gugel@unife.it (I.G.), ilenia.gugel@unife.it (Ilenia.G.), stefania.costa@unife.it (S.C.), anna.baldisserotto@unife.it (A.B.), erika.baldini@unife.it (E.B.), smanfred@unife.it (S.M.) and silvia.vertuani@unife.it (S.V.)

* Correspondence: stefania.costa@unife.it, filippo.marchetti@unife.it

† Irene Gugel and Erika Baldini equally contributed to this work

Supplementary materials

Parameter	Unit	Typically
Dry substance	%	38-52
Ash	%	7-12
pH	-	3.5-4.5
Protein*	%	36-49
Copper	mg/kg	5
Iron	mg/kg	250
Lactic acid*	%	22.50
Manganese	mg/kg	40
Nitrogen from aminoacids*	%	2.50
Phosphorus*	%	3.50
Reducing sugars*	%	2
Total nitrogen*	%	7.40
Zinc	mg/kg	200
Choline	mg/kg	1990
Myo-inositol	mg/kg	3250

* based on dry basis

Table S1. Chemico-physical specifications of corn-steep liquor (data provided by the supplier Cargill s.r.l.).

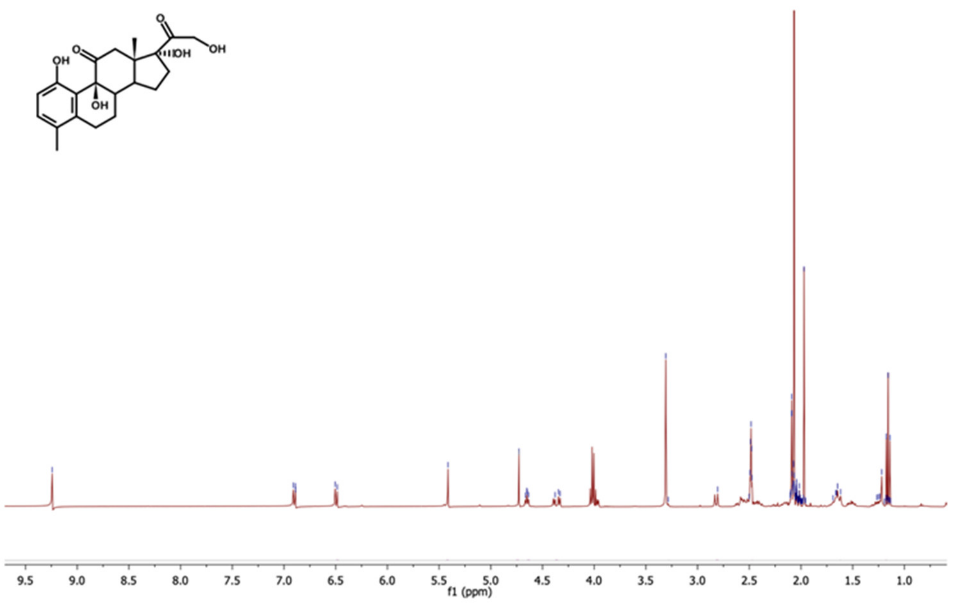

A

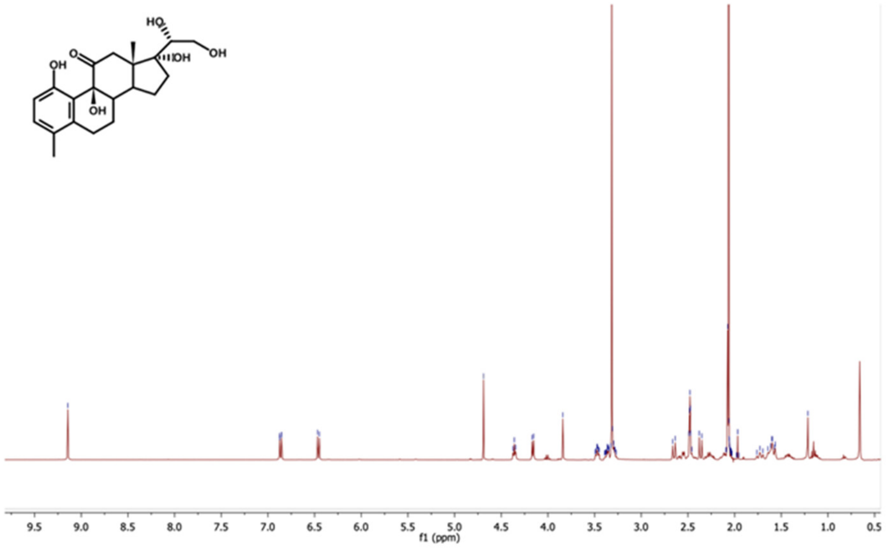

B

Figure S1 ^{1}H-NMR spectra of SCA (A) acquired at 400 MHz in DMSO-d_6 and ^{1}H-NMR spectra of SCB (B) acquired at 400 MHz in DMSO-d_6.

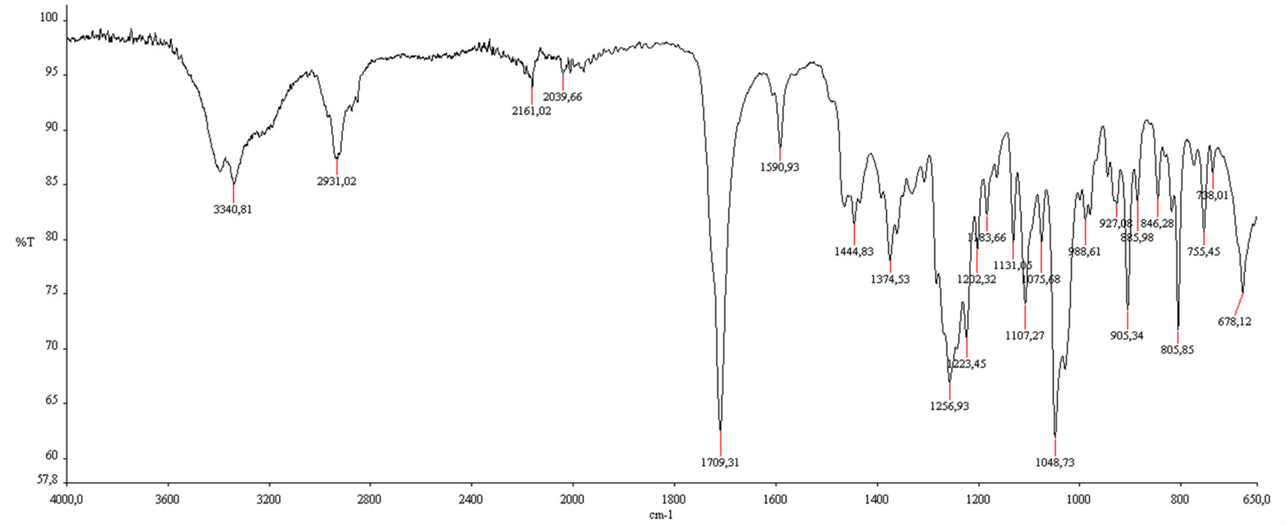

A

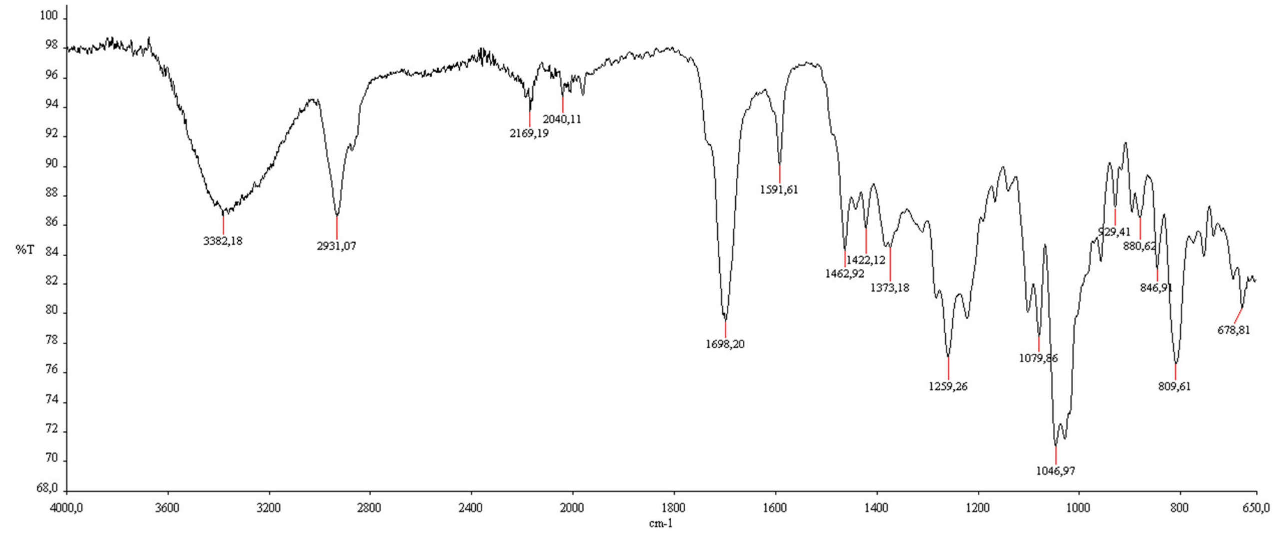

B

Figure S2. FTIR spectra of SCA (A) and SCB (B) derived from bioconversion of cortisone in UOLM.
